# Time to find out what Brexit really means

**DOI:** 10.1007/s12399-021-00844-x

**Published:** 2021-04-26

**Authors:** Anand Menon, Matthew Bevington

**Affiliations:** 1 UK in a Changing Europe, 22 Kingsway, WC2B 6LE London, UK; 2 Global Counsel, 5 Welbeck Street, W1G 9YQ London, UK

**Keywords:** Brexit, UK, EU, Politics, Trade, Brexit, Großbritannien, EU, Handel

## Abstract

Brexit is done. Yet there remain more questions than answers. A new relationship has been established, but the functioning of the two new treaties in practice remains uncertain. The EU remains a contentious issue in UK politics and will continue to hang over political debates, especially in Scotland. The UK and EU have to define a new relationship with a political interest in seeing the other fail. Ultimately, what Brexit is likely to mean is more Brexit, both in terms of ongoing UK-EU conflicts and in deep divisions over the EU in UK politics.

## Abstract (de)

Der Brexit ist vollzogen. Dennoch bleiben mehr Fragen als Antworten. Die neue Beziehung fußt auf zwei Abkommen, deren Funktionieren in der Praxis ungewiss bleibt. Die EU bleibt ein Streitthema der britischen Politik und der politischen Debatten, vor allem in Schottland. Die EU und das Königreich müssen ein neues Verhältnis zueinander aufbauen, haben aber ein politisches Interesse daran, den anderen scheitern zu sehen. Letztlich wird der Brexit wahrscheinlich noch mehr Brexit bedeuten, sowohl bei Konflikten zwischen dem Vereinigten Königreich und der EU als auch bei der politischen Spaltung der britischen Politik bei EU-Themen.

## Introduction

And so Brexit rolls on. Yes, Boris Johnson campaigned, first for the Conservative Party leadership, then during the General Election of 2019, on a pledge to *Get Brexit Done*. Yet the direct implications of the treaty signed between the UK and the EU, as well as the indirect consequences of both Brexit and the tortuous process that has unfolded since the referendum of 2016, mean that Brexit will continue to haunt the UK and its politics for some time to come.^1^ Just over a year since that election, Johnson has indeed delivered Brexit, first by taking the UK out of the EU and then by leaving the EU single market with a trade agreement. Moreover, his overwhelming victory in the December 2019 election meant an end to the parliamentary stalemate of Theresa May’s hung Parliament and a return to the strong, single-party government that long characterised British politics. Yet for all those achievements, a remarkable level of uncertainty remains. There are still more questions than answers. How will the EU develop without the UK as a member state? How will the UK decide to use its new-found autonomy? What impact will both of those have on the arrangements in the recently agreed Trade and Cooperation Agreement? What impact will all this have on the UK union? In what follows, we discuss the dilemmas that Brexit continues to present to the British government and for UK-EU relations. We also try to offer some insight for an EU audience as to how to understand the thinking of the British government and how the EU fits into the wider domestic political landscape.

## Brexit is over. Long live Brexit

The agreement and ratification of the two treaties that now form the basis of UK-EU relations mark the end of negotiations that took the best part of four years to complete. Negotiating teams on both sides have been dismantled. Michel Barnier will soon head back in French politics after a brief period overseeing the ratification of the deal for the Commission in the European Parliament. David Frost has taken up a new post as the Prime Minister’s Brexit and International Policy Representative. Their respective exits are emblematic of the fact that we are entering a new phase of the Brexit process. And yet UK-EU negotiations are far from over. As recent weeks have made all too clear, managing the implementation of the Northern Ireland Protocol alone is going to be a time-consuming and politically combustible business. The Joint Committee, which manages the Withdrawal Agreement, and the Partnership Council, which does the same for the Trade and Cooperation Agreement (TCA), are the pinnacle of structures comprising over 30 committees, working groups and other bodies (Thimont Jack and Rutter 2021). These will make for intense ongoing interactions. Moreover, there are already several negotiations and review dates in the diary. On fisheries, although quotas were decided in the TCA, the UK still needs to agree the total allowable catch with the EU in their respective waters. These will be annual negotiations starting this year, mirroring those the UK took part in as a member state. The whole TCA agreement will be reviewed in 2025. Then there are disputes. The aborted attempt by the Commission to trigger the emergency Article 16 clause in the Northern Ireland Protocol shows how unexpectedly conflicts can arise. The two sides remain deeply integrated, economically, socially and, thanks to the two UK-EU agreements, legally (see below). This deep interdependence means that further periodic disputes are inevitable. Nor will Brexit disappear from British politics either. It will continue to cast a long shadow over party politics in the UK, as we discuss further below. But perhaps more fundamentally, as attention turns from the EU to the UK union itself, Brexit will loom large over independence debates in Scotland (as well as in Wales and Northern Ireland). The Scottish Parliament elections in May will be a catalyst, whatever the result. A pro-independence majority of the Scottish National Party (SNP) and the Greens, both of whom were strongly anti-Brexit, will likely trigger an attempt by the Scottish government to legislate for a second independence referendum. Constitutionally, formal powers sit with the UK government, but it is unclear whether the Scottish government can call a merely advisory referendum. That, partly, is the point – to escalate the issue into the courts, and ultimately the UK Supreme Court, to continue to apply pressure to the government in Westminster and keep independence a salient issue, even in the midst of a pandemic. And Brexit will be a central issue in the debates to come. For all the talk in UK politics about Brexit representing *the will of the people*, that will was different in Scotland. Not only that, but the UK government systematically ignored the wishes of the Scottish government when negotiating its Brexit deal, and pursued a hard form of Brexit that rubbed salt into the wounds of many Scottish voters. Johnson himself is also wildly unpopular north of the border while Scottish First Minister Nicola Sturgeon, at least for the moment, appears unassailable. The UK government faces the difficulty of explaining why, in light of sustained polling showing a small majority for independence, the public should not have another say. The Scottish National Party are not without their difficulties either. Just as many British Conservatives will find themselves accused of hypocrisy on Scottish independence – pointing to the economic costs, not least from trade barriers with the rest of the UK – the SNP will be accused of hypocrisy for downplaying them. Recent economic analysis shows that, at least where trade is concerned, the impact on Scotland would be two to three times larger than the impact of Brexit on the UK (Huang et al. 2021). Moreover, rejoining the EU would not offset these additional trade barriers with the rest of the UK, which is even more important for Scottish trade than the EU is for the UK. In sum, the independence debate, which is likely to rage through the rest of this parliament, goes hand-in-hand with the Brexit outcome. And Brexit will also continue to play out in the politics of Northern Ireland. The Northern Ireland Assembly will be asked in 2024 to give its consent to the arrangements set out in the Northern Ireland Protocol, voting every four or eight years thereafter. If consent is refused, the Protocol will cease two years later, necessitating urgent UK-EU discussions on alternatives. In Northern Ireland, one way or another, it will be *Brexiternity*. The Democratic Unionist Party (DUP) has already begun to campaign to get rid of the Protocol, prompted, not least, by its own falling poll ratings (DUP 2021; LucidTalk 2021). There could not have been a bigger gift to the DUP than the Commission’s blunder in appearing to trigger Article 16 in an attempt to monitor the export of vaccines to the UK. They had been calling for the Prime Minister to trigger the clause and take unilateral action of his own, but he had demurred. He now has to explain why he cannot trigger it when the Commission were apparently willing to do so freely. Brexit has already had a profound impact on politics in Northern Ireland. Across the political spectrum, there is agreement that the east-west trade arrangements between Great Britain and Northern Ireland are not functioning well. And this while there are multiple grace periods in place. We have not yet reached peak disruption. So, while we have moved into a new phase of the process, Brexit continues to be a major issue that will linger in British politics for a long time to come, perhaps indefinitely. Both the Conservatives and the opposition Labour Party want to focus on other priorities, but they may have little choice. And as we have seen in recent weeks, there remain political opportunities to capitalise on any future difficulties in the relationship.

## Britannia (not quite) Unchained

**Fig. 1:**
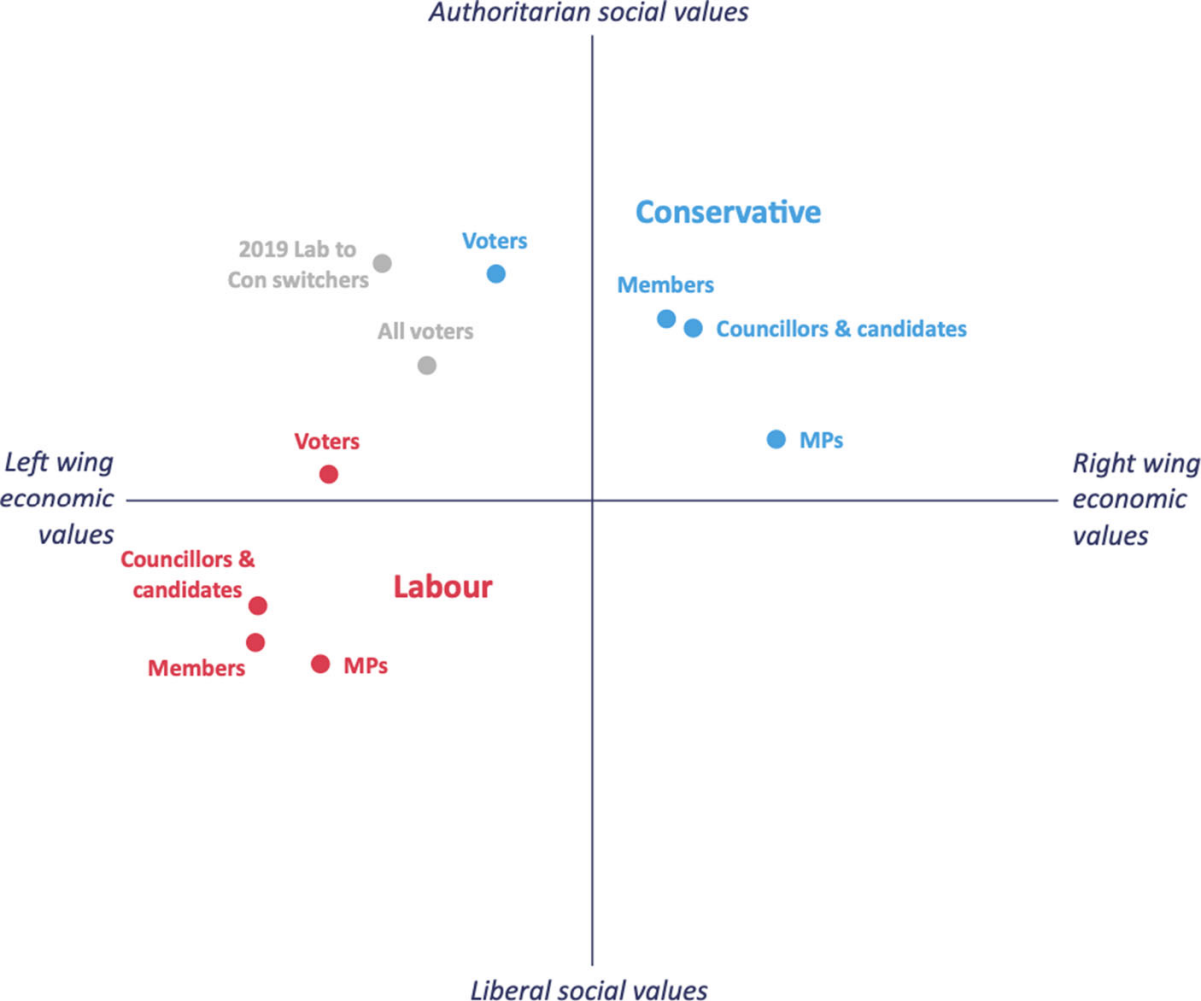
Voters are more aligned with Labour on economic values and with the Conservatives on social values. Economic and social values of Conservative and Labour MPs, councillors and candidates, members, and voters, 2020. (Source: ESRC Party Members Project survey, YouGov, fieldwork Dec 2019; UK in a Changing Europe MPs survey, Ipsos Mori, fieldwork, Jan–Feb 2020; BES Internet panel Wave 14, fieldwork Jun 2017, Wave 17, fieldwork Nov 2019, and Wave 19, fieldwork Dec 2019)

Now that the UK has left the EU and the transition period has ended, UK politicians need to decide what the domestic implications of Brexit will be. More specifically, they will have to figure out what they want to do with the *control* that they have won back from Brussels. Remarkably, almost five years after the referendum, there remains a significant degree of uncertainty over the substantive ambitions of the government. It is in the midst of launching a rash of consultations – from gene editing to subsidies – with business and interest groups to figure out what it can now do. It has long been an article of faith for Conservative Eurosceptics that a fundamental problem with the EU resides in its tendency to over-regulate. Many took their inspiration from Margaret Thatcher’s speech to the College of Europe in Bruges, when she stressed free markets and wider choice, and the fact that the goal should “not be more and more detailed regulation from the centre; it should be to deregulate and to remove the constraints on trade” (Thatcher 1988, as cited in Margaret Thatcher Foundation 2021). Of course, Thatcher was making this argument in support of the creation of the single market, which she saw as based on the desire to create a Europe “open to enterprise”. Her successors, however, came to view things rather differently. In 2013, for instance, two leading Eurosceptics – Bill Cash and Bernard Jenkin – argued that the benefits of the single market were not worth the costs (Cash and Jenkin 2013). Throughout the Brexit process, and particularly following the election of Johnson as leader of the Conservative Party, British negotiators insisted that they would reject any deal that left the UK subject to EU law and the jurisdiction of the European Court of Justice. When the Prime Minister’s Chief Negotiator, David Frost, delivered a speech at another Belgian university in February 2020, he underlined that it “is central to our vision that we must have the ability to set laws that suit us […] it is the point of the whole project” (Frost 2020). The aim of cutting loose from EU-imposed regulation was, it seemed, alive and well. Indeed, to such a point that Johnson was willing to accept a border in the Irish Sea if that were the price for regulatory autonomy for Great Britain. And yet early signs are that the government is perhaps not as committed to the kind of deregulatory agenda one might have assumed would be front and centre of the plans of a post-Brexit Conservative government. As Jill Rutter has pointed out, regulatory freedom does not necessarily mean lower levels of regulation (Rutter 2020). There are two reasons why the government might feel constrained when it comes to embarking upon the bonfire of regulations that some of its supporters still clearly favour (Zorzut 2021). The first relates to the EU itself. Although the UK negotiated the freedom to diverge from EU regulations and paid the price in terms of market access costs, this independence is still significantly constrained. In the key areas of financial services and data, decisions about future relations were not taken in the formal trade negotiations but, rather, will be settled by the EU itself when it considers granting equivalence and adequacy decisions respectively covering those areas. This has significant implications for future regulatory freedom in that EU decisions will hinge in part on perceptions of what form future UK regulation might take. In both cases in future, the EU reserves the right to revisit its decisions should circumstances demand. The Chancellor, Rishi Sunak, has assarbiured businesses that the UK intends to grant better access to EU markets than is provided for under the TCA (Walker 2020). At the same time, however, he has insisted that the UK can do things differently post-Brexit, not least by making the City of London the most attractive place to list companies in the world. It remains to be seen whether the EU will be happy to grant market access via equivalence to a competitor economy openly promising to make use of regulatory freedom to compete with its own financial centres. More broadly, the TCA includes provisions designed to limit the extent of regulatory competition. Where significant divergences occur between EU and UK law that have a material impact on trade or investment, one party may impose sanctions within 19 days of notifying the other, or 49 days if the matter is referred to arbitration and there has been no ruling. According to one analysis, the terms of the treaty are vague enough to imply that any regulatory difference that affects competitiveness “will likely meet the threshold to allow the EU to take action” (Chalmers 2020). By extension, if the UK disagrees, it can take proportionate counteraction. And so, such conflicts may easily escalate. The central Brexit compromise – between autonomy and trade – will continue to haunt UK decision makers. Free to diverge from EU regulations, the UK government may still face retaliation from Brussels should it choose to exploit this right. The EU is one reason why Johnson might hesitate before making use of his hard-won regulatory freedom. Domestic politics is another. A preference for deregulation has long been a hallmark of the Conservative Party. But that was then. The Brexit process had the effect, among other things, of altering the social bases of party competition in the UK. To simplify only somewhat, what Johnson succeeded in doing in the December 2019 election was pulling together an overwhelmingly pro-Leave coalition (Skinner et al. 2019). The price of constructing an electoral alliance held together by values and social identity, however, is that Conservative voters are not necessarily united when it comes to economic questions. Fig. 1, taken from a UK in a Changing Europe report, illustrates the point graphically (Bale et al. 2020, p. 14).

Conservative MPs are far more right wing on economic issues than most voters and particularly those who switched from Labour to the Conservatives in 2019. Intuitively, it is easy to see how traditional relatively well-off Conservative voters from the English shires might have different economic preference to those new Conservatives in northern, post-industrial Red Wall seats. These divisions are also apparent in Parliament. Having purged the Conservative Parliamentary Party of Brexit non-believers – many of whom, on economics, were more traditional free-market Tories – Johnson has reformed his party as well. There remain some traditional Conservatives, wedded to a small state, deregulation and low taxes. But there is also the Northern Research Group of Tory MPs, comprising more than 50 members that is committed to *levelling up* the British economy by addressing the geographic inequalities that blight the country. For this latter group, the idea that the post-Brexit priority is to cut regulation, including workers’ rights, is far from compelling. Domestically too, then, the Prime Minister faces pressures when it comes to his approach to post-Brexit regulation. For all the years of Conservative backbench dislike of EU red-tape, it may well be that Brexit Britain, albeit formally freed from the shackles of EU law, keeps many of the same rules in place. The Conservative Party that has come out of the Brexit process is substantially different to the one that entered it.

## Remember, Brussels is the capital of Belgium

One of the fundamental arguments made by the pro-Brexit side during the referendum and afterwards was that the EU was increasingly irrelevant and, in fact, holding back its member states. Instead, the argument went, the UK should focus its attentions on the more economically dynamic parts of the world, namely the Asia-Pacific region. Moreover, given the rise of China, it is that region where the most important geopolitical action will take place in the coming decades. In a fast-moving, multi-polar world, the UK needed the freedom to navigate between fast-shifting alliances. In this context, it was argued, there would be more opportunities outside of Europe than within it. As appealing as such ideas might be, the stubborn facts of geography are a heavy counterweight. Prior to the pandemic, our ability to move quickly across the world had never been greater. Advanced communication technologies mean that many multinational businesses operate 24 hours a day across multiple time zones. On the face of it, geography would seem to have become less relevant. Yet when it comes to trade it remains the single best predictor of trade patterns. Even in terms of mobility, British citizens go to Europe – for work, holiday and to study – vastly more often than they go anywhere else. Geography may be less important in theory, but in practice it remains decisive. The UK has sought to reassert its nationhood since leaving the EU, and even before. This has manifested itself recently in a refusal to grant the EU ambassador to the UK full diplomatic status because the EU, the UK government argues, is an “international organisation”, not a nation state. There have been other hints of this thinking as well. In response to a UK Foreign Affairs Committee report on future UK global strategy, the government said: “The Government agrees with the Committee’s view on the importance of sustaining the UK’s strong, historic ties with *European states* [emphasis added]” (House of Commons 2021, p. 8). The European Union itself did not merit a single mention. The UK embassy to the EU has also been downgraded. The diplomatic rank of the new Head of Mission, Lindsay Croisdale-Appleby, is a grade below that of his predecessor. The deputy ambassador role has also been scrapped, and the size of the mission itself is being steadily reduced. This may be deliberate provocation, but the thinking is rife with contradiction. The case for leaving the EU was based partly on the argument that its supranational institutions were too powerful. And yet, now, they barely warrant recognition. The UK’s former Chief Negotiator turned Brexit Policy Representative liked to antagonise his EU counterparts by describing the EU as “your organisation” (Boffey 2020) during negotiations. Yet in a speech in February 2020, Frost himself described the EU as a “new governmental system overlaid on an old one, purportedly a Europe of nation states, but in reality the paradigm of a new system of transnational collective governance” (Frost 2020). The upshot is that those who hoped for a more pragmatic approach from the British in future may be sadly disappointed. The EU remains ripe for political point-scoring in the UK. The UK government is understandably keen to show that Brexit has been – and will be – a success. It has scored an early victory on that front with the Covid-19 vaccine rollout, and may score another if it is able, as a result, to open up its economy and society faster than its EU neighbours. This reflects a zero-sum view of UK-EU relations. The success of Brexit will be measured against the performance of the EU, and especially its larger member states. Their difficulties relative to the UK will be weaponised to show the comparative benefits of leaving the EU. The key question from a British perspective is whether the UK can effectively pursue its interests working exclusively with member states, or whether it also needs comprehensive engagement with the EU institutions. Contrary to the government’s approach, the evidence suggests the latter. Take Sanctions. These are typically decided at EU level. As the sanctions against the Belarusian regime in 2020 showed, the UK may be nimbler alone, but it is also less impactful. It had wanted to coordinate with the EU, but slow decision-making by the latter meant the UK eventually went ahead without it. There are, of course, opportunities for fruitful bilateral interactions, which must be seized. The E3 arrangement with France and Germany has deepened in recent years as they have sought to co-manage the Iran nuclear deal and influence other global events. For other member states keen to strengthen relations with the UK, bilateral channels are much more likely to be successful than EU-level proposals. If you ask an EU member state minister to describe Brussels, they might say the home of the EU institutions. If you ask a British minister, they’re more likely to say the capital of Belgium. This, in essence, captures the difference in thinking.

## Keeping Brexit done

Looking forward, it is interesting and important to reflect in more detail on the potential continued interplay between UK-EU relations and domestic politics. Given, as we’ve pointed out, that Johnson won the 2019 election on the back of a pledge to *get Brexit done*, it might be expected that it is in his interests to stop mentioning the “B word” now that a deal has been signed with the EU. This, however, may not be the case. What will be the case is that, for all the omissions in the TCA, the current government is highly unlikely to seek to resume negotiations. As Frost said in Parliament in February, “I don’t think there are any new things that need to be done by way of additions to the agreement” (Frost, as cited in Parliamentlive.tv 2021). There seems little prospect, for instance, that Johnson will come to regret the absence of any agreement on foreign and defence policy to the point where he attempts to initiate formal talks about them. Nor will the scale of east-west frictions between Great Britain and Northern Ireland change the fundamental calculation that he made in 2019. More likely, Johnson will seek to use the provisions in the UK-EU agreements to ease any frictions and, as he showed with the Internal Market Bill in 2020, may even go beyond them if politically useful. The deal, in other words, will remain the deal. Although the UK government looks unlikely to restart formal negotiations with the EU, we might expect it periodically to pick fights with Brussels (and vice versa), as it has done over the status of the EU Ambassador in the UK. The explanation relates back to our earlier discussion of the nature of the political coalition the Conservatives assembled in December 2019. An electoral alliance united by values is one that can be appealed to by, inter alia, conflict with the EU. And there is another incentive. The opposition Labour Party in some ways represents the mirror image of the governing party in that it is relatively united on economic policy but divided when it comes to social values. Although the seats it lost to the Conservatives in 2019 had overwhelmingly backed Leave, most of Labour’s voters are Remain supporters. This puts the party in a difficult situation, which is why the leadership is keen to avoid any discussion of Brexit. Labour leader Keir Starmer has not only decided that, if elected Prime Minister, he will not seek to make major changes to the UK-EU relationship negotiated by Johnson (Elgot 2020), but also abandoned a commitment to freedom of movement he made when running for the leadership (Sparrow 2021). His hope is that putting the Brexit issue to bed will allow him to focus attention on economic issues over which it is the governing party is divided. All too aware of the divisions within the opposition when it comes to Brexit, the Prime Minister therefore has an interest in keeping the issue alive until the next election. It is reasonable for him to make use of the five-yearly review of the TCA to raise the danger of Labour seeking to renegotiate the treaty, stressing the need to *keep Brexit done* as a way not only of uniting his own electorate but also of potentially exposing the divisions in the Labour camp.

## Partners and rivals

All this provides a glimpse of what UK-EU relations will be like in the medium term. There are severe limits on the extent to which relations can be *normalised*, partly because the two sides will have to define a new normal. If we look to the EU’s relations with close partners and neighbours, such as Switzerland or Turkey, these are hardly conflict-free relationships. And there are reasons to think that UK-EU relations could be even more tense given the sheer scale of economic interactions and the nature of politics in the UK. Seemingly innocuous policy decisions made by either side could easily escalate into substantial conflicts. And the potential for tit-for-tat is almost endless in the TCA. This is all the more so given the fact that for some on the EU side at least, it is important that Brexit comes to be seen as a failure. The Commission, member states and MEPs may be satisfied, even pleased, with the outcome of negotiations. They have managed to impose a high price on access to the single market and severely constrained the access that has been granted. If this is the outcome for one of the EU’s largest member states wanting to leave, what access would smaller member states with less bargaining power end up with? Nevertheless, whatever the UK does, whether consciously or not, it is unavoidably a model for life outside the EU. And this will continue to be a matter of concern to both the Commission and member state governments, particularly those confronted with populist opponents. Yet beyond the obvious tensions and rivalries that will be an inevitable part of UK-EU relations in future, the UK has compelling reasons to want to have productive relations with both the EU and its member states. The UK’s core economic and strategic interests remain in Europe, and its surrounding regions. Whether on trade, investment, migration, security or defence, the biggest risks lie close to home. Think back to the eurozone crisis. David Cameron fought tooth and nail to keep the UK out of eurozone bailouts, but also vigorously encouraged integration efforts among those countries. At the infamous Fiscal Compact summit in December 2011, he recognised “the crisis in the Eurozone is having a chilling effect on Britain’s economy” (GOV.UK 2011). The relationship is not zero sum, and as well as being competitors both sides have an overwhelming interest in their mutual success. For a politician with the right instincts, this message could easily be sold domestically in the UK. However, Johnson may not be that kind of politician. The British public remains deeply divided on the merits of Brexit, even if many Remain supporters have come to accept the post-Brexit reality (Curtice 2021). Over the long term, the demographic trends may also favour a progressively more pro-EU population. Somehow the pro-Brexit side of the argument still finds itself on the defensive, needing to prove the worth of the decision, even though it is signed, sealed and delivered (Ford 2020). The 2024 election slogans have almost already written themselves: Vote Conservative, prevent Labour taking us back into the EU. The emphasis, at least for the next few years, may be more on seeing each other as rivals than partners.

## Conclusion

The UK has left the EU, but it will not be able to leave the EU behind. Geography, economics and geopolitics mean that the UK will always have a strategic interest in the EU, not just individual member states. Equally, the UK remains strategically important for the EU. The bloc has to balance seeking to circumscribe any unfair competitive advantages the UK might seek to develop while also wanting to retain the UK as a close ally and partner on the world stage. For neither will the post-Brexit settlement be one that they are completely at ease with. The UK has got the economic settlement of Canada (arguably a slightly worse one) with the political settlement of Switzerland. Its access to the EU single market is highly constrained, yet the two UK-EU treaties mean that the two sides are going to be in near-continuous negotiation of one kind or another for the foreseeable future. And as these negotiations drag on, so too can we expect that the direct and indirect impacts of Brexit on British policy, politics and the UK polity itself will continue to make themselves felt.

## Footnotes

1 For an understanding of the EU perspective on Brexit and future UK-EU relations, see also the article by Birgit Bujard and Wolfgang Wessels (2021) in this issue.
